# The prognostic significance of GRB7 protein expression and localization in human breast and ovarian cancers

**DOI:** 10.18632/oncotarget.27593

**Published:** 2020-06-16

**Authors:** Anke Vermehren-Schmaedick, Paulette Mhawech-Fauceglia, Byung S. Park, Tanja Pejovic, Shiuh-Wen Luoh

**Affiliations:** ^1^ Hospital & Specialty Medicine, VA Portland Health Care System, Portland, OR, USA; ^2^ Translational Oncology Program, Knight Cancer Institute, Oregon Health & Science University, Portland, OR, USA; ^3^ Division of Hematology and Medical Oncology, Knight Cancer Institute, Oregon Health & Science University, Portland, OR, USA; ^4^ Department of Anatomic Pathology, Aurora Diagnostics/Sonic Healthcare, Las Vegas, NV, USA; ^5^ Biostatistics Shared Resource, Knight Cancer Institute, Oregon Health & Science University, Portland, OR, USA; ^6^ School of Public Health, Oregon Health & Science University - Portland State University, Portland, OR, USA; ^7^ Division of Gynecologic Oncology, Department of Obstetrics and Gynecology, Oregon Health & Science University, Portland, OR, USA

**Keywords:** GRB7 expression, breast cancer, ovarian cancer, immunohistochemistry, prognosis

## Abstract

**Objective:** To study GRB7 protein expression in normal human tissues and breast and ovarian cancers, and determine its clinical significance.

**Results:** GRB7 protein was expressed in multiple tissues, including myoepithelial cells of normal breast and fibroadenoma. Cytoplasmic GRB7 expression was seen predominantly in HER-2 positive and, to a lesser extent, triple negative breast cancer. Membrane localization of GRB7 was present in a subset of breast cancers with high cytoplasmic GRB7 expression. Univariate and multivariate analysis found that cytoplasmic GRB7 expression was associated with a negative progesterone receptor status, while membrane GRB7 expression was associated with a negative axillary nodal status. Membrane associated GRB7 expression was present in a subset of ovarian cancers with high cytoplasmic GRB7 expression. Membrane GRB7 expression displayed a trend towards improved recurrence free survival (RFS). Landmark analysis suggested an RFS advantage for ovarian cancers that had GRB7 membrane expression and survived beyond 27 months; GRB7 membrane expression in two or more cores (out of three) predicted an improved RFS. Membrane expression of GRB7 protein was observed in breast cancer cell lines with high GRB7 protein expression *in vitro*.

**Conclusion:** GRB7 protein membrane expression may be associated with a better prognosis in breast and ovarian cancers. The favorable prognostic value of GRB7 protein membrane expression and its underlying mechanism is worthy of further investigation.

**Methods:** Immunohistochemistry of normal human tissues, breast tissues of various pathologies, and clinically annotated ovarian cancers was performed to correlate the patterns of GRB7 expression with biomarkers or clinical outcome.

## INTRODUCTION

Growth factor receptor-bound protein-7 (GRB7) protein is a 532 amino-acid adaptor molecule that lacks intrinsic enzymatic activity, but mediates signal transduction from multiple cell surface receptors to specific downstream signaling pathways inside the cell. The GRB7 protein contains an amino-terminal proline-rich region, a central GM (Grb/Mig) segment with a pleckstrin homology (PH) domain to bind phospholipids which could recruit GRB7 to the plasma membrane, and a carboxyl-terminal src-homology 2 (SH2) domain involved in the recruitment of host proteins to activated upstream target receptors [1, 2]. Consequently, GRB7 has been shown to be involved in regulation of cell proliferation, cell migration and invasion in the case of carcinoma cells, and tumor formation [3–5].

The GRB7 gene is located in close proximity to the HER-2/erbB2 gene, forming the HER-2-amplicon core, and is commonly, though not always, co-amplified and overexpressed with HER-2 in human breast cancers [6–10]. Although the consensus is that HER-2 positive breast cancer is generally associated with poor clinical outcome, we and others have found that GRB7 protein over-expression is a stronger independent adverse prognostic factor than HER-2 over-expression [9, 11]. The significance of GRB7 function is further supported by the laboratory findings that GRB7 over-expression facilitates HER-2 mediated signaling, breast cancer cell proliferation, and tumor formation in an animal model [4]. Knock down of GRB7 expression on the other hand, reduced the growth of HER-2 positive breast cancer cells in culture and as tumor xenografts in animal models [5, 12]. In addition, GRB7 expression is found to be an adverse prognostic factor for triple negative breast cancer (TNBC), both *in vivo* and *in vitro* [13, 14]. Results of past studies of GRB7 expression in ovarian cancer with both RT-PCR and IHC, show that expression of GRB7, and its variant GRB7v, has been associated with high grade ovarian cancer [15]. The prognostic significance of GRB7 expression has otherwise not been reported in ovarian cancer.

GRB7 protein expression has been reported in cytoplasm, focal adhesions, stress granules, and the nucleus (reviewed in [2]). Deletion of the SH2 domain of GRB7 ablates the localization of GRB7 in focal contacts [16], and interaction of the PH domain with phospholipid may recruit GRB7 to the cell membrane [17]. GRB7 is an integral component of the stress granules and a key mediator in the formation of the nuclear-cytoplasmic export complex [18]. A specific sequence has been identified with significant similarity to nuclear localization signals that may mediate the GRB7 enhancement in the nucleus [19]. A membrane accentuated GRB7 protein localization has not been reported in the past.

GRB7 overexpression was found to be significantly correlated with metastatic cancers. In esophageal and pancreatic carcinomas, GRB7 was positively correlated with the presence of lymph node metastases [3, 20]. High GRB7 expression was also found to be associated with clinical progression of hepatocellular carcinoma [21]. In addition, GRB7 showed clinic-pathological significance in cervical cancer, while high GRB7 levels were correlated with age, tumor size and stage, serosal invasion, lymph node metastasis, lower overall survival rate [22]. Aberrant upregulation of GRB7 expression was also strongly associated with decreased survival in patients with breast cancer [11], and a multi-variate analysis revealed that GRB7 protein overexpression was an independent adverse prognostic factor for breast cancer-free interval [9]. Moreover, GRB7 expression has also been found to be a significant predictive factor to drug response and recurrence after cancer treatment, as it is exemplified in patients with operable TNBC where high GRB7 RNA and protein expression levels were associated with an elevated risk of recurrence in patients with breast cancer [13, 14].

In the current study we report the tissue distribution of GRB7 protein by IHC in normal human tissues, including benign breast diseases. Additionally, we performed GRB7 IHC on both breast and ovarian cancer TMAs. We find GRB7 membrane expression in both ovarian and breast cancers that are associated with high cytoplasmic expression of GRB7. Membrane expression of GRB7 protein is observed in breast cancer cell lines with high GRB7 protein expression by IF and IHC. GRB7 membrane expression is associated with less nodal involvement in breast cancer and a trend towards improved recurrence free survival in ovarian cancer.

## RESULTS

### GRB7 expression in normal human tissues

GRB7 has distinctive expression patterns among multiple human tissues as shown by immunohistochemistry (IHC). GRB7 is abundantly expressed in tissues such as bone marrow, salivary glands, brain (cerebellum and cerebral cortex), heart, prostate and kidney (cortex), yet the amount of GRB7 is variable in liver, skeletal muscle, endometrium, stomach and colon. Lower GRB7 expression is seen in breast, fallopian tubes and pancreas, while almost no GRB7 expression was detected in the esophagus, lung, ovary or spleen (see Table 1 for a complete list of GRB7 expression levels, and Supplementary Figure 1 for representative IHC images).

**Table 1:** GRB7 expression in normal human tissues

GRB7 3+	GBR7 2+	GRB7 1+	GRB7 0	Unclassified
Bone marrow [3, 3]	Adrenal gland [2, 1, 2]	Bladder [1, 1, 2]	Eye [0,0]	Kidney (medulla) [3, 1]
Salivary gland [3, 3]	Ureter [2, 2]	Breast [1, 2, 1]	Esophagus [0,0,0]	Pituitary gland [1, 3]
Cerebellum [3, 3, 3]		Fallopian tube [1, 2, 1]	Lung [0,0,0]	
Cerebral cortex [3, 3, 3]		Stomach [1, 3, 1]	Ovary [0,0,0]	
Heart [3, 3, 3]		Small intestine [2, 1, 1]	Skin [0,0,0]	
Kidney (cortex) [3, 3, 3]		Colon [3,0,1]	Spinal cord [0,0]	
Liver [2, 3, 3]		Rectum [1, 2, 1]	Spleen [0,0,0]	
Prostate [3, 3, 3]		Pancreas [1, 1, 1]	Thymus [0,0,0]	
Skeletal muscle [3, 2, 3]		Placenta [1, 1, 1]	Thyroid [0,1,0]	
Tonsil [2, 3, 3]		Testis [2, 1, 1]	Uterus (cervix) [0,0,0]	
Uterus (endom) [2, 3, 3]				

Scoring of GRB7 expression in 35 tissues following immunohistochemistry (Tissue Micro Arrays obtained from Pantomics, Cat# MNO1021; 28 tissues in triplicates, 7 tissues in duplicates). Each column lists tissues based on their GRB7 expression levels (3+, 2+, 1+, and 0 = no-expression). Numbers in brackets indicate the GRB7 expression level for each replicate.

**Figure 1:**
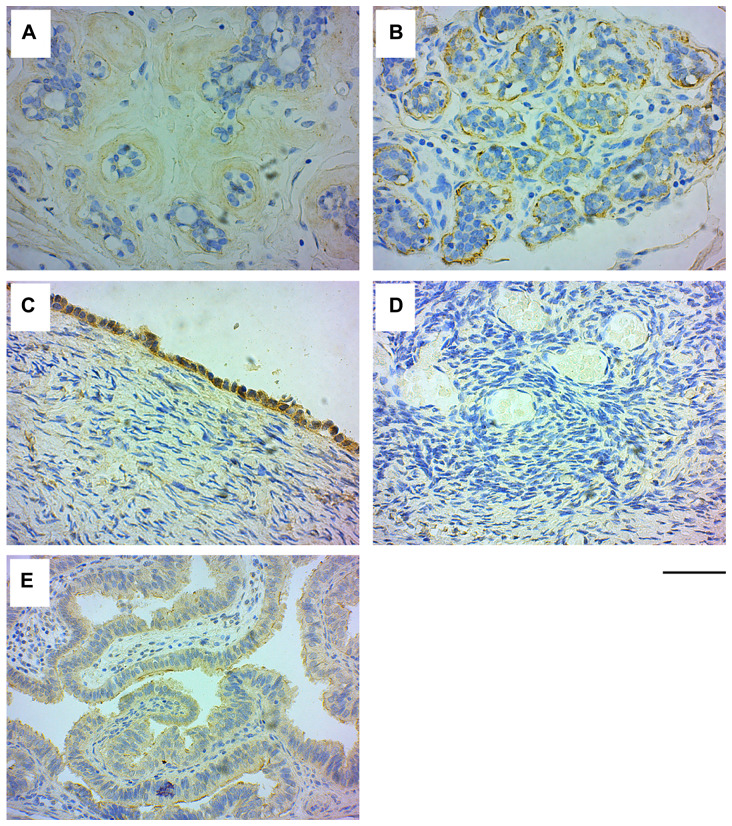
GRB7 expression in normal breast and ovarian tissues. Representative images of normal tissues stained with the GRB7 antibody (brown) and Hematoxylin (blue). (**A**) Normal breast tissue showing no GRB7 expression in the mammary ducts. (**B**) Normal breast tissue showing high GRB7 expression (3+) in the myoepithelial cells of mammary ducts. (**C**) Normal ovarian surface epithelium showing strong cytoplasmic GRB7 (3+) expression. (**D**) Normal ovarian tissue showing no GRB7 expression in the stroma. (**E**) Normal fallopian tubes with weak GRB7 (1+) expression. All images were taken with a 40× objective (scale bar 50 μm).

IHC analysis of normal breast tissue samples (*n =* 30) with GRB7 antibodies revealed that in 33% (*n =* 10; mean age 38.50 ± 10.66 years) of the cases, GRB7 expression was found in the myoepithelial cells surrounding the mammary gland ducts, while there was no observable GRB7 staining in the rest of the samples (*n =* 20; mean age 42.15 ± 7.39 years) (see Figure 1A, 1B).

IHC analysis of the normal ovarian tissue samples showed that GRB7 was highly expressed in the cytoplasm of surface epithelial cells, while the ovarian parenchyma and stroma were negative for GRB7 staining. In addition, the fallopian tubes showed weak cytoplasmic GRB7 expression in the columnar epithelium lining the tubes (see Figure 1C–1E).

### GRB7 expression in normal breast and breast cancer TMAs

Commercially obtained TMA slides containing normal and cancerous breast tissue samples were screened. Of the 505 sample cores, we removed 21 samples due to duplication, 27 due to lost tissue, and 8 due to sections being different from breast glandular tissue (for more details see Supplementary Figure 2). From the remaining 449 sections, 30 sections were of normal breast tissue, and 419 sections showed various types of breast cancer histologies: 7.16% (*n =* 30) were benign, 80.91% (*n =* 339) were invasive ductal carcinoma (IDC), 6.68% (*n =* 28) were ductal carcinoma *in situ*, 3.10% (*n =* 13) were invasive lobular carcinomas, and the remainder (2.15%, *n =* 9) included atypical hyperplasia, mucinous adenocarcinoma, adenoid cystic carcinoma and Paget’s disease.

Overall, GRB7 expression in normal breast and breast cancer tissues was found mainly in the cytoplasm of epithelial cells, with high expression (score of 3+) in 19.15% of the samples, medium expression (score of 2+) in 15.14% of the sections, weak expression (score of 1+) in 25.17% of the samples and the absent of staining (score of 0) in the rest (40.53%). Interestingly, in some cases, GRB7 was accentuated in the membrane in addition to being found in the cytoplasm (52.33%, 17.65% and 2.65% in the 3+, 2+ and 1+ cases respectively). For a more detailed summary of the GRB7 expression levels and location for the different histologies, see Table 2.

**Table 2:** Summary of GRB7 expression in breast tissue cores

	GRB7 3+		GRB7 2+		Grb7 1+		GRB7 0	Total
	c	c/m	c	c/m	c	c/m	c	
Normal tissue	2^*^	0	1^*^	0	7^*^	0	20	30
Benign (fibroadenoma)	4^*^	0	1^*^	0	3^*^	0	7	15
Benign (fibrocystic changes)	0	0	0	0	0	0	15	15
Invasive ductal carcinoma	27	42	48	11	94	3	114	339
Ductal carcinoma *in situ*	5	3	5	0	2	0	13	28
Invasive lobular carcinoma	1	0	1	0	3	0	8	13
Atypical hyperplasia	2	0	0	0	1	0	2	5
Mucinous adenocarcinoma	0	0	0	0	0	0	2	2
Adenoid cystic carcinoma	0	0	0	0	0	0	1	1
Paget’s disease	0	0	0	1	0	0	0	1
**TOTAL**	41	45	56	12	110	3	182	449

A list of normal and cancerous breast tissues indicating the GRB7 expression levels and frequencies. Under each GRB7 staining intensity (3+, 2+, 1+, 0) are the number of cases that show cytoplasmic staining only (c) or cytoplasmic plus membrane accentuation (c/m). (^*^) denotes GRB7 expression in the myoepithelial cells.

### GRB7 expression in benign fibroadenoma and fibrocystic changes

**Figure 2:**
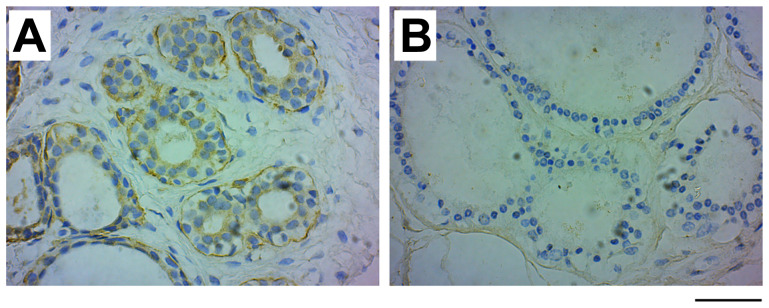
GRB7 expression in benign fibroadenoma and fibrocystic changes. Examples of images of benign breast cancer tumors stained with the GRB7 antibody (brown) and Hematoxylin (blue). (**A**) Benign fibroadenoma tissue showing high GRB7 expression (3+) in myoepithelial cells. (**B**) Benign fibrocystic changes showing no GRB7 expression (0). Images were taken with a 40 × objective, scale bar = 50 μm).

Our study includes two types of benign breast diseases, fibroadenoma, a localized non-cancerous tumor, and fibrocystic breast changes, characterized by an overgrowth of cells that line the milk ducts, increased fibrous tissue and formation of cysts. Interestingly, about half (53.3%) of the fibroadenoma samples (*n =* 15; mean age 29.50 ± 11.35 years) expressed GRB7 in the myoepithelial cells, while no GRB7 expression was seen in the fibrocystic changes samples (*n =* 15; mean age 24.00 ± 8.91 years) (see Figure 2).

### GRB7 expression in invasive ductal carcinoma of the breast

**Figure 3:**
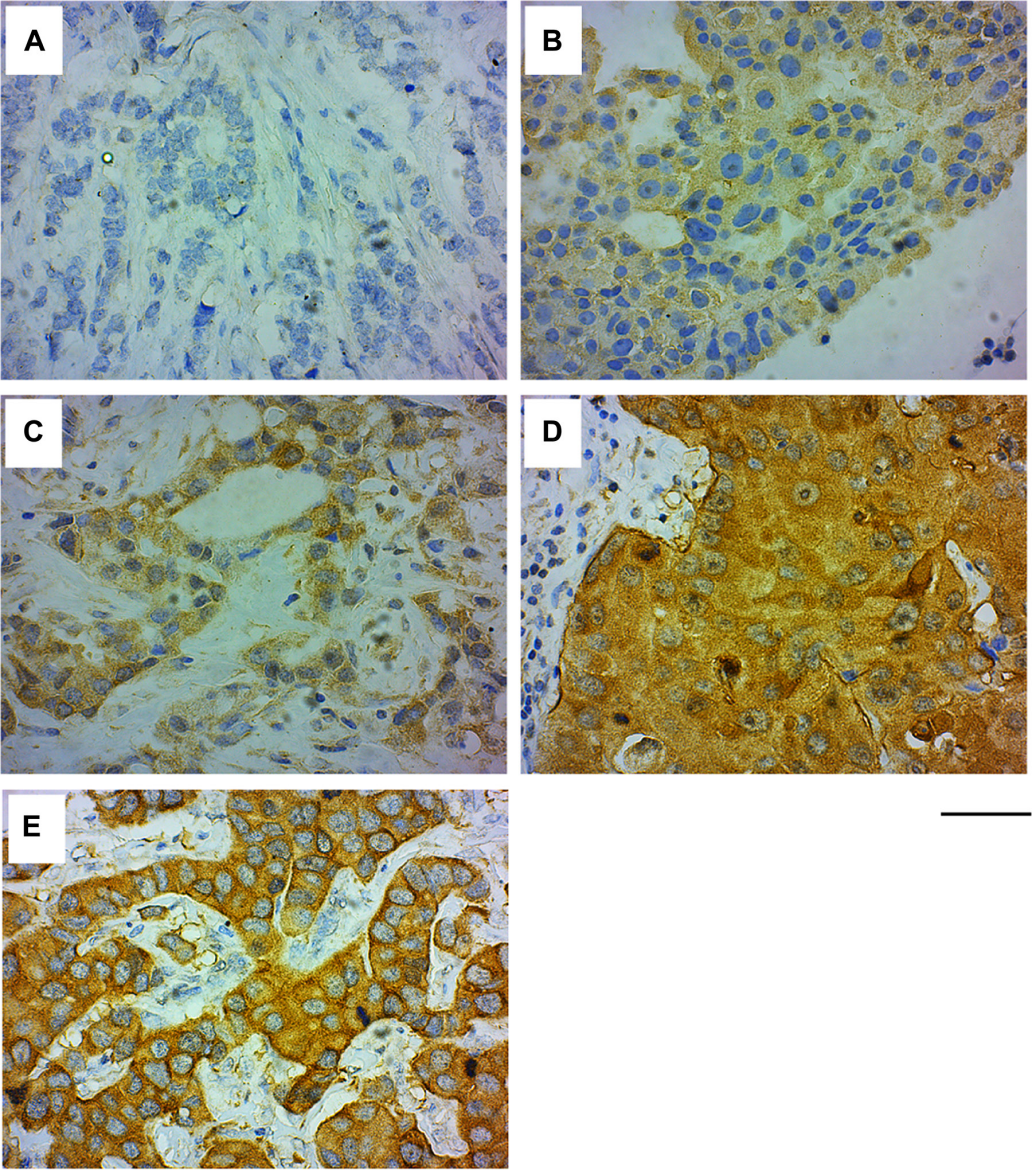
GRB7 expression and localization in invasive ductal carcinoma (IDC) of the breast. Representative images of IDC of the breast tissues stained with the GRB7 antibody (brown) and Hematoxylin (blue). (**A**) IDC showing no GRB7 expression (0). (**B**) IDC showing weak cytoplasmic GRB7 expression (1+). (**C**) IDC showing medium cytoplasmic GRB7 expression (2+). (**D**) IDC showing strong cytoplasmic GRB7 expression (3+). (**E**) IDC showing strong cytoplasmic GRB7 expression with membranous accentuation (3+). Images were taken with a 40 × objective, scale bar = 50 μm.

We next examined the GRB7 expression levels and localization within the cell, and correlated these results with clinical factors in the invasive ductal carcinoma (IDC) samples. The age range of the study subjects (*n =* 339) is 18 to 86 years, with an average age of 49.32 ± 11.39, and a median of 49 (see Table 3). IHC analysis of IDC samples showed that GRB7 protein was mainly expressed in the cytoplasm of cells ranging from weak (1+) to medium (2+) to strong (3+) (see Figure 3A–3D), with membrane accentuation seen primarily in high GRB7 expressers (3+) (Figure 3E and Table 2).

**Table 3:** Relationship between patient age for 339 patients with invasive ductal carcinoma of the breast and GRB7 expression

GRB7	*N*	Mean age	Median age	StdDev	Min	Max	95% CI (for mean)
0–1+	210^a^	49.99	48	11.83	18	86	(48.38–51.59)
2+–3+	128	48.23	49	10.58	27	73	(46.39–50.08)
Total	338	49.32	49	11.39	18	86	(48.10–50.54)

GRB7 protein expression levels in the cytoplasm is shown as low (no staining to weak staining, 0 to 1+) *vs.* high (medium to strong staining, 2+ to 3+). (a) One data point was removed due to missing age information (for GRB7–1+).

Table 4 describes the association of GRB7 protein expression levels in the cytoplasm, shown as low (no staining to weak staining, 0 to 1+) *vs.* high (medium to strong staining, 2+ to 3+), with breast cancer risk factors that are known to be associated with clinical outcomes. Overexpression of GRB7 was found to be associated with HER-2, ER and PR markers (*p <* 0.0001, *p <* 0.0001 and *p <* 0.001, respectively), while no association was observed for age, tumor size and lymph node involvement. Breast cancer risk factors were then further analyzed using a multivariate logistic regression method in order to identify independent prognostic factors (Table 5). Here we found that HER-2 positive (3+) status has a 20.106 higher odds having high GRB7 expression as HER-2 negative (0–1+) (*p*-value < 0.0001), and that PR negative status has a 1.818 times larger odds having high GRB7 expression as PR positive status (*p*-value = 0.0326).

**Table 4:** GRB7 cytoplasmic expression and clinico-pathological characteristics in 339 patients with invasive ductal carcinoma of the breast

			**GRB7 expression**				**Chi-squared *p*-value**
Clinical factor	Categories	0–1 (low)	%	2–3 (high)	%	Total	
Age	<50	115	54.76	67	52.34	182	0.6653
	≥50	95	45.24	61	47.66	156	
	total	210		128		338	
Tumor size	<2 cm	12	5.69	5	3.91	17	0.4664
	≥2 cm	199	94.31	123	96.09	322	
	total	211		128		339	
Nodes	Neg	148	70.14	88	68.75	236	0.7870
	Pos	63	29.86	40	31.25	103	
	total	211		128		339	
HER-2	Neg	130	62.20	7	5.47	137	**<0.0001**
	Equiv	18	8.61	13	10.16	31	
	Pos	61	29.19	108	84.38	169	
	total	209		128		337	
ER	Neg	101	48.56	91	72.22	192	**<0.0001**
	Pos	107	51.44	35	27.78	142	
	total	208		126		334	
PR	Neg	114	54.81	103	81.75	217	**<0.001**
	Pos	94	45.19	23	18.25	117	
	total	208		126		334	

Descriptive frequency and univariate chi-square *p*-values for GRB7 protein expression levels in the cytoplasm shown as low (no staining to weak staining, 0 to 1+) *vs.* high (medium to strong staining, 2+ to 3+). Neg = negative, Pos = positive, Equiv = equivocal. Not all data points were available for all patients.

**Table 5:** Multivariate logistic regression analysis in 331 patients with invasive ductal carcinoma of the breast using GRB7 cytoplasmic expression (low 0 - 1+ *vs.* high 2+ - 3+) as the response variable

		95% Confidence interval		
Effect	Odds ratio	Lower	Upper	*p*-value
Age (<50 *vs*. ≥50)	1.057	0.691	1.619	0.7975
Tumor size (<2 *vs*. ≥2)	0.972	0.618	1.526	0.9004
Nodes (N *vs*. P)	1.113	0.413	2.999	0.8321
HER-2 (N *vs*. P)	20.106	11.574	34.928	**<0.0001**
HER-2 (Equiv *vs*. P)	3.929	1.792	8.615	**0.0006**
ER (N *vs*. P)	1.174	0.699	1.971	0.5445
PR (N *vs*. P)	0.550	0.317	0.952	**0.0326**

N = negative, P = positive, Equiv = equivocal. Not all data points were available for all patients. Due to the missing, total of 331 (not 339) were used for multivariate logistic model.

In order to examine the biological significance of GRB7 membrane expression, we studied possible associations between cytoplasmic GRB7 protein expression with membrane accentuation with breast cancer risk factors. Table 6 shows that membrane localization is significantly associated with lymph node involvement, HER-2 and PR status (*p*-values of 0.0269, 0.0002 and 0.0458, respectively). Multivariate logistic regression showed that negative lymph node status has 2.294 times higher odds of having cytoplasmic GRB7 expression and membrane accentuation as positive lymph node status (*p*-value = 0.0341), and that HER-2 positive status has 10.880 times larger odds of having cytoplasmic GRB7 expression and membrane accentuation as HER-2 negative (*p*-value = 0.0017) (Table 7).

**Table 6:** GRB7 protein location (cytoplasmic *vs.* cytoplasmic + membrane accentuated) and clinico-pathological characteristics in 225 patients with invasive ductal carcinoma of the breast

			GRB7 location				Chi-squared
Clinical factor	Categories	Cyto	%	Cyto + Memb	%	Total	*p*-value
Tumor size	<2	9	5.33	2	3.57	11	0.5978
	≥2	160	94.67	54	96.43	214	
	total	169		56		225	
Nodes	Neg	109	64.50	45	80.36	154	**0.0269**
	Pos	60	35.50	11	19.64	71	
	total	169		56		225	
HER-2	Neg	47	27.98	2	3.57	49	**0.0002**
	Equiv	18	10.71	4	7.14	22	
	Pos	103	61.31	50	89.29	153	
	total	168		56		224	
ER	Neg	97	58.08	38	70.37	136	0.1142
	Pos	70	41.92	16	29.63	86	
	total	168		54		222	
PR	Neg	113	67.26	44	81.48	157	**0.0458**
	Pos	55	32.74	10	18.52	65	
	total	168		54		222	

Descriptive frequency and univariate chi-square *p*-values for patients with cytoplasmic expression (Cyto) *vs.* patients with cytoplasmic expression and membrane accentuation (Cyto + Memb). Positive GRB7 expression includes 1+, 2+ and 3+. Neg = negative, Pos = positive, Equiv = equivocal. Not all data points were available for all patients.

**Table 7:** Multivariate logistic regression analysis in 221 patients with invasive ductal carcinoma of the breast using GRB7 location (cytoplasm *vs.* cytoplasm + membrane accentuation) as the response variable

		95% Confidence interval		
Effect	Odds ratio	Lower	Upper	*p*-value
Tumor size (N *vs*. P)	0.966	0.154	6.061	0.9706
Nodes (<2 *vs*. ≥2)	0.436	0.203	0.940	**0.0341**
HER-2 (N *vs*. P)	10.880	2.447	48.376	**0.0017**
HER-2 (Equiv *vs*. P)	4.332	0.808	23.222	0.0870
ER (N *vs*. P)	1.196	0.508	2.814	0.6815
PR (N *vs*. P)	0.608	0.233	1.587	0.3092

N = negative, P = positive, Equiv = equivocal. Not all data points were available for all patients. Due to the missing, total of 221 (not 225) were used for multivariate logistic model.

When we looked at the possible association between GRB7 expression levels and membrane staining, we found that there was a significant association (chi-square test *p*-value < 0.0001, Table 8) between GRB7 expression levels and location, but the simple kappa coefficient estimate is only 0.35 (StdEr 0.05, 95% CI 0.26–0.45). This is in part due to the observation that only a percentage of breast cancer samples with GRB7 high expression have membrane expression.

**Table 8:** Association between GRB7 expression (low/high) and location (cytoplasm/membrane) in 225 patients with invasive ductal carcinoma of the breast

Categories		GRB7 location			
		Cyto	Cyto + Memb	Total	*p*-value
**GRB7 Expression**	Low (1+)	94 (55.62)	3 (5.36)	97	**<0.0001**
	High (2+ – 3+)	75 (44.38)	53 (94.64)	128	
	total	169	56	225	

Cyto = cytoplasm alone, Cyto + memb = cytoplasmic with membrane accentuation.

When we analyzed the sections with triple negative breast cancer (TNBC, *n =* 48), we found 4 with medium (2+) GRB7 expression, 10 with weak (1+) GRB7 expression, and 34 samples with no GRB7 expression (0). These results are consistent with our prior findings that a fraction of breast cancers that are TNBC have GRB7 expression [10, 14]. In addition, we also looked at the HER-2 negative population (HER-2 = 0–1+, regardless of ER and PR scoring, *n =* 137), and found 1 with strong (3+) and membrane-accentuated GRB7 expression, 6 with medium (2+) GRB7 expression, 42 with weak (1+) GRB7 expression, and 88 samples with no GRB7 expression (0).

### Cytoplasmic GRB7 expression in ovarian cancer

It has been previously shown that GRB7 expression is associated with high-grade ovarian cancer [23], but the correlation between GRB7 overexpression and clinical outcome is not known. To look at this possible correlation, we used TMAs with ovarian cancer tissue samples from 187 patients, each in triplicates. Of the 187 patient samples, we removed 14 patient samples due to loss of all three tissue replicates. For the remaining 173 patients, we had their recorded age at the time of diagnosis, date of diagnosis, primary treatment, date of surgery, type of ovarian cancer, date of recurrence, date of last follow up and disease status.

A summary of the Clinical parameters analyzed for this cohort of patients is summarized in Supplementary Table 1. The age of diagnosis range of the study subjects is 20 to 89 years, with an average age of 61.14 ± 12.31, and a median of 62 years. Patients who had neo-adjuvant chemotherapy as the primary treatment represented 19.65% (*n =* 34/173), while 80.35% (*n =* 139/173) had adjuvant chemotherapy. The histology types of the ovarian cancers were 21.39% (*n =* 37/173) for non-high-grade serous carcinoma (non-HGS), and 78.61% (*n =* 136/173) for high-grade serous ovarian cancer (HGSOC), as previously reported [15].

**Figure 4:**
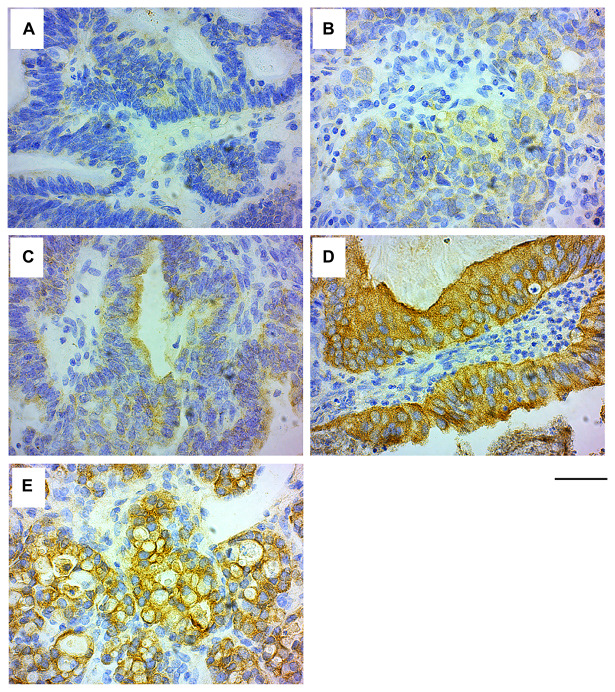
GRB7 expression in ovarian carcinoma cells. Representative images of high grade ovarian cancer tissues stained with the GRB7 antibody (brown) and Hematoxylin (blue). (**A**) No GRB7 expression (0). (**B**) Weak cytoplasmic GRB7 expression (1+). (**C**) Medium cytoplasmic GRB7 expression (2+). (**D**) Strong cytoplasmic GRB7 expression (3+). (**E**) Strong cytoplasmic GRB7 expression with membranous accentuation (3+). Images were taken with a 40 × objective, scale bar = 50 μm.

Similar to the GRB7 expression in breast IDC sections, IHC analysis of the ovarian tissues showed that GRB7 was mainly expressed in the cytoplasm of cells, ranging from no GRB7 expression (26.59%, 46/173) to weak (1+, 32.37%, 56/173) to medium (2+, 19.08%, 33/173) and strong (3+, 21.97%, 38/173) GRB7 expression, for a total of 73.41% patients expressing GRB7 in the cytoplasm (see Figure 4A–4D). Membrane accentuation was seen primarily in high GRB7 expressers (47.37%, 18/38), followed by medium and low GRB7 expressers (24.24%, 8/33 and 8.93%, 5/56 respectively, Figure 4E). When we look at the 389 cores left after the IHC staining from the 173 unique patients in this TMA (130 cores were lost before the staining), 146 cores did not express GRB7 (37.53%), and 243 (62.47%) expressed different levels of GRB7 expression: 61/243 (15.68%) was strong, 52/243 (13.37%) was medium and 130/243 (33.42%) was weak. When we looked at the possible association between GRB7 expression levels and membrane staining, we found that there was a significant association between GRB7 expression levels and location (chi-square test *p*-value < 0.0001, Table 9), but there was only 63% agreement between the GRB7 expression and GRB7 location (simple kappa coefficient estimate of 0.23; StdEr 0.05, 95% CI 0.12 to 0.33). As with IDC of the breast, membrane expression of GRB7 is primarily seen in ovarian cancer samples with high GRB7 cytoplasmic expression (3+) but only a fraction of ovarian cancer samples with high cytoplasmic expression of GRB7 protein (3+) has membrane accentuation.

**Table 9:** Association between GRB7 expression (Low/High) and location (cytoplasm/membrane) in 243 cores from 173 patients with ovarian cancer

**Categories**		**GRB7 location**			
		Cyto alone	Cyto + Memb	Total	*p*-value
**GRB7 expression**	Low (1+)	119 (60.10)	11 (24.44)	130	**0.000015**
	High (2+ – 3+)	79 (39.90)	34 (75.56)	113	
	total	198	45	243	

Cyto = cytoplasm alone, Cyto + Memb = cytoplasmic with membrane accentuation.

The clinical events in our ovarian cancer analysis are defined as Recurrence Free Survival (RFS, the time from diagnosis to recurrence, progression, or death due to any cause, whichever occurs first) and Overall Survival (OS). An overview of the characteristics of these ovarian cancer patients can be found in the Supplementary Table 2, and their survival curves in Supplementary Figure 3 and Supplementary Table 3.

When we looked at the cytoplasmic expression of GRB7, we found no significant differences in the length of RFS nor OS for expression (1+, 2+, 3+) *vs*. no expression (0) (log-rank test *p*-value of 0.6687 and 0.7850 respectively, see Supplementary Figure 4 and Supplementary Table 4A). In additional analyses, no differences were seen when we compared high GRB7 expression (3+) *vs*. medium (2+), weak (1+) or none (0) (log-rank test *p*-value of 0.7484 and 0.7166 respectively), or when we compared high GRB7 expression (3+) with less GRB7 expression as a group (0 to 2+) (log-rank test *p*-value of 0.4190 and 0.8321 respectively). See Supplementary Table 4B–4C for more details.

### GRB7 membrane-association is of clinical significance for ovarian cancers

**Figure 5:**
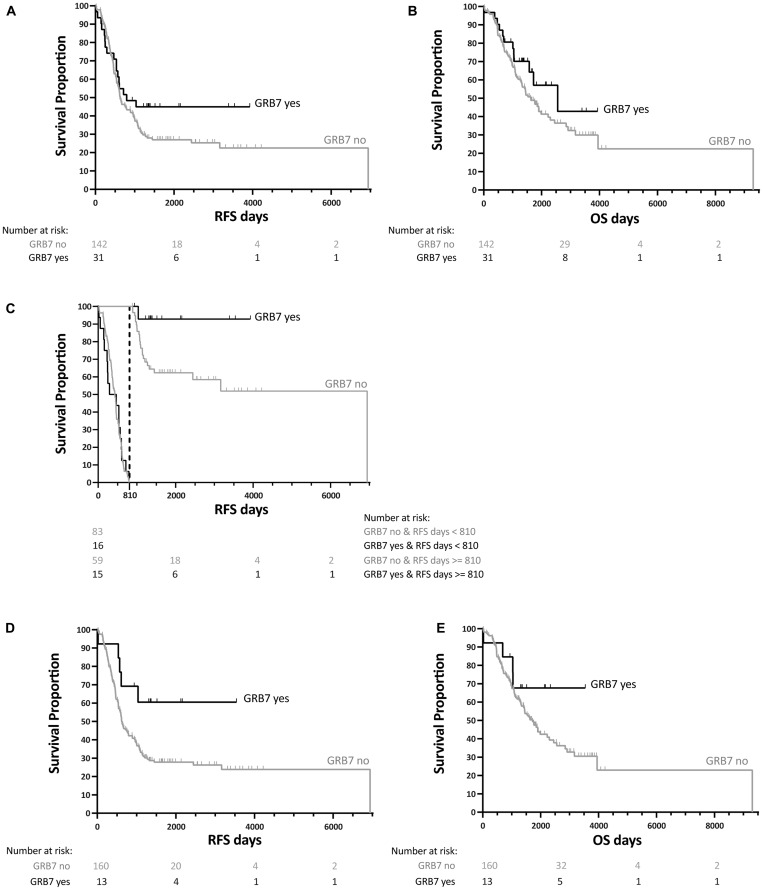
Kaplan-Meier survival analysis for GRB7 membrane-associated staining from 173 patients with ovarian cancer. (**A**) Recurrence Free Survival (RFS) and (**B**) Overall Survival (OS) curves for each patient showing GRB7 membrane-associated expression shown as yes (black curve)/no (gray curve) for each patient). (**C**) Landmark analysis for RFS (shown in (A) using 810 days (27 months) as the cut off mark (dotted lined). (**D**) RFS and (**E**) OS curves for patients with at least two out of three tissue cores with GRB7 membrane-associated expression (black curve) *vs.* one or no GRB7 membrane-associated expression (gray curve). All survival curves were generated after dichotomization of GRB7 membrane-associated expression (black curves, “GRB7 yes”) or no GRB7 membrane-associated expression (gray curve, “GRB7 no”), obtained by IHC. *p*-values were calculated by log-rank tests.

We then analyzed the membrane-associated GRB7 expression, and found that there was no significant difference in RFS nor OS (log-rank test *p*-value of 0.1709 and 0.1952 respectively). On a closer look of the data, there appeared to be a delayed effect for GRB7 membrane expression and RFS, and performing a landmark analysis using 27 months (810 days) as our cut off mark, patients that survived after 27 months had a significantly better RFS if they also had GRB7 membrane-associated expression than if they did not show membrane staining (log-rank test *p*-value of 0.8081 before the 27 months, *p*-value of 0.0353 for after the 27 months), suggesting a result of clinical significance. No significant results were found for GRB7 membrane-associated staining for OS. See Figure 5 and Table 10A for more information.

**Table 10:** Survival analysis of patients with GRB7 membrane-associated staining in ovarian cancer

A	# of + cores	# of events				Median survival			Mean survival		Log-rank test *p*-value
		Total	Event	Censored	Censor %	Estimate	Lower 95% CI	Upper 95% CI	Mean	SD	
RFS	0	142	102	40	28.17	630.00	540.00	892.00	2107.84	254.49	0.1709
	≥1	31	17	14	45.16	797.00	533.00	.	702.52	67.39	
	all	173	119	54	31.21	648	575	947	2323.23	239.99	
OS	0	142	80	62	43.66	1616.00	1292.00	2223.00	3259.43	485.98	0.1952
	≥1	31	12	19	61.29	2554.00	1570.00	.	1870.68	172.65	
	all	173	92	81	46.82	1758.00	1411.00	2299.00	3427.58	492.31	

B	# of + cores	# of events				Median survival			Mean survival		Log-rank test *p*-value
		Total	Event	Censored	Censor %	Estimate	Lower 95% CI	Upper 95% CI	Mean	SD	
RFS	<2	160	114	46	28.75	619.00	540.00	797.00	2166.89	241.00	**0.0373**
	≥2	13	5	8	61.54		570.00		850.69	95.47	
	all	173	119	54	31.21	648	575	947	2323.23	239.99	
OS	<2	160	88	72	45	1719.00	1330.00	2223.00	3310.74	479.76	0.2217
	≥2	13	4	9	69.23		1036.00		935.88	89.59	
	all	173	92	81	46.82	1758.00	1411.00	2299.00	3427.58	492.31	

(A) Membrane staining, when analyzed as no GRB7 staining (0) *vs*. at least 1 core positive per patient (≥1), had no significant effect on RFS nor OS. (B) Membrane staining, when analyzed as fewer than 2 cores positive for GRB7 staining (<2) *vs*. at least 2 positive cores per patient (≥2), shows a significant lengthening of the RFS, but not of the OS. *p*-values given are log-rank test.

In addition, when we analyzed the results of GRB7 membrane staining comparing RFS of patients with two or three membrane expressing cores (out of three) with those with fewer or none expressing, we found that there was an overall significant lengthening of the RFS in these patients (log-rank test *p*-value 0.0373). There was no effect on the OS (log-rank test *p*-value 0.2217, see Table 10B).

### GRB7 expression in breast cancer cell lines

**Figure 6:**
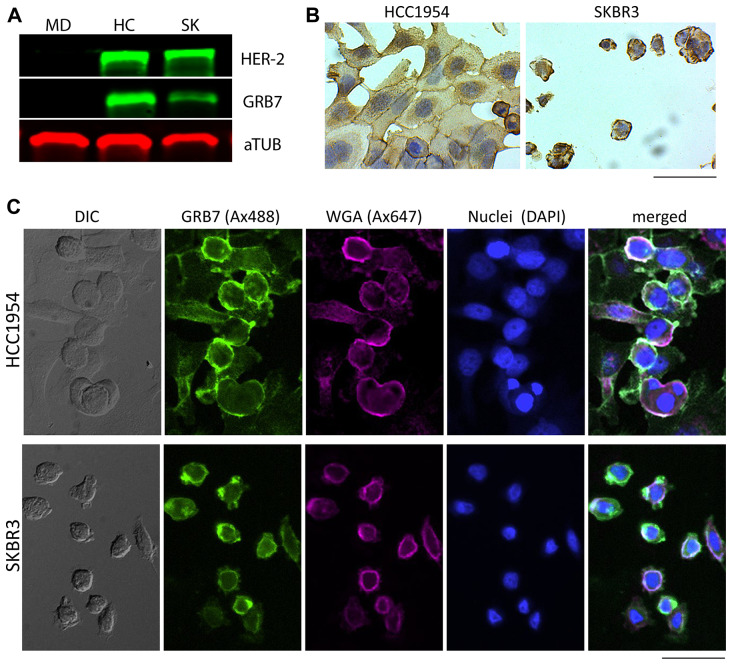
GRB7 expression levels and localization of breast cancer cell lines (MDA-MB-231, HCC1954, and SKBR3). (**A**) Western blot showing GRB7 expression levels (middle) panel using total protein lysates (10 μg/lane). Alpha-tubulin (aTUB, bottom panel) was used as internal reference, HER-2 was used to show co-amplification of HER-2 and GRB7 as expected (top panel). MD = MDA-MB-231, HC = HCC1954, SK = SKBR3. (**B**) Representative images of breast cancer (HCC1954, SKBR3) cell lines stained (IHC) with the GRB7 antibody (brown, developed using HRP-DAB) and nuclei (Hematoxylin, blue). Images show cells with strong cytoplasmic GRB7 expression with membranous accentuation. (**C**) Representative images of breast cancer (HCC1954, SKBR3) cell lines stained (IF) with the GRB7 antibody (green), WGA cell membrane marker (magenta), nuclei (blue), and a merged image from all three channels, showing GRB7 and WGA co-localization in white (most right). A DIC image is shown on the most left. Images were taken with a 20 × objective, scale bar = 100 μm.

We explored the possibility that breast and ovarian cancer cell lines also display GRB7 membrane-associated staining. For this we analyzed MDA-MB-231, HCC1954, SKBR3 breast cancer cell lines. As expected, MDA-MB-231 cells did not express GRB7, while HCC1954 and SKBR3 cell lines did on Western analysis (Figure 6A). Interestingly, on immunohistochemistry and immunofluorescence analysis, we found analysis (Figure 6A). Interestingly, on IHC analysis, we found that for HCC1954 and SKBR3 breast cancer cell lines there were varying degrees of GRB7 membrane-associated expression (Figure 6B, 6C).

## DISCUSSION

GRB7 is a signal transduction molecule and has been implicated in the biology and clinical behaviors of multiple cancers including breast and ovarian cancers [2]. Though frequently amplified and over-expressed concurrently with HER-2 in breast cancers with chromosome 17q11-12 amplification, we and others have shown that GRB7 expression is an independent adverse prognostic factor for breast cancer, including the triple negative breast cancer subtype [9, 13, 14]. GRB7 expression is also associated with high grade ovarian cancer [15].

In this study, we performed IHC on TMAs from normal tissues and demonstrate GRB7 protein expression in multiple tissues from multiple origins. We found that in normal breast tissues, GRB7 protein expression is present in the myoepithelial cells. Myoepithelial cells separate the luminal epithelial layer from the surrounding basement membrane and can contribute to differentiation, proliferation and polarity of the luminal epithelial cells; and there is evidence for myoepithelial cells as promoters or suppressors of cancer cell invasion [24]. The biological significance of GRB7 expression in myoepithelial cells is currently unknown. In benign breast diseases, GRB7 expression is present in the myoepithelial cells of the fibroadenoma but not fibrocystic disease. In normal ovarian tissues, GRB7 expression is present in the ovarian epithelium.

In breast cancers, a high (3+) or a moderate (2+) level of GRB7 protein expression in the cytoplasm is present in 19.72% and 16.90% of breast cancers, respectively. A high level of GRB7 expression (3+) is highly associated with HER-2 protein over-expression (3+), but as previously reported by us and others only a fraction of breast cancer specimens with HER-2 over-expression (3+) has high GRB7 protein expression (3+). A low (1+) to moderate (2+) level of GRB7 protein expression is noted in triple negative breast cancers as described earlier [9].

Most notably, we find that when GRB7 protein is expressed in the cell membrane, it is often associated with high (3+) GRB7 protein cytoplasmic expression, though 3 + GRB7 cytoplasmic staining also occurs in the absence of membrane accentuation. Univariate followed by multivariate analysis finds that GRB7 cytoplasmic expression is associated with high HER-2 protein over-expression and negative progesterone receptor status. GRB7 membrane expression however is associated with HER-2 protein over-expression and negative axillary node status. The negative prognostic value of GRB7 cytoplasmic expression was consistent with prior findings [9, 11]. The different clinical implications for membrane versus cytoplasmic expression of GRB7 protein is a novel finding and may reflect the outcome of interaction of GRB7 protein with diverse signaling molecules at different cellular locations.

Membrane expression of GRB7 protein has been observed in ovarian cancer samples. As with breast cancer, membrane expression of GRB7 protein is present in ovarian cancer samples with high GRB7 expression (3+) in the cytoplasm but only a fraction of ovarian cancer specimens with high cytoplasmic expression of GRB7 protein (3+) exhibit GRB7 membrane expression. GRB7 protein and RNA expression have been associated with high grade ovarian cancers [15]. We find in the current study, that membrane expression of GRB7 protein is associated with a trend towards an improved recurrence free survival in our primary analysis. Cytoplasmic expression of GRB7 protein on the contrary is not associated with clinical outcome in our ovarian cancer cohort. We performed additional exploratory analyses. Landmark analysis finds a significantly improved recurrence free survival in a subset of patients that survived beyond 27 months. Moreover, among ovarian cancer samples with two or three, rather than only one core that display membrane expression on the TMA, there is an improvement in recurrence free survival. As with breast cancer, membrane expression of GRB7 protein is associated with a favorable prognosis. The improved recurrence free survival associated with the GRB7 membrane staining in the ovarian cancer may be due to a better prognosis and/or increased response rate to the systemic therapy such as platinum based chemotherapy. This warrants further investigation.

Though intracellular localization of GRB7 protein had been the subject of prior studies, membrane accentuation had not been previously reported. This could be due to the antibody reagents used in prior studies, or overexpression of epitope tagging of GRB7 protein in experimental systems [15, 17]. Epitope tagging may interfere with proper trafficking of GRB7 proteins in the cells. In this study we confirmed the membrane accentuation of GRB7 protein expression in human breast cancer cell lines with HER-2 and GRB7 protein over-expression.

In conclusion, our study finds that membrane-affiliated expression of GRB7 may be associated with a better prognosis in both breast and ovarian cancers. GRB7 protein expression in the cytoplasm and membrane in fact, carries the opposite prognostic significance for breast cancer. The above may reflect the outcome of diverse signaling events in the cells. Further study is warranted to understand the signaling complexities that GRB7 emanates from different cellular locations. This knowledge is essential for development of GRB7 directed therapeutics and understanding of its involvement in breast and ovarian cancer biology.

## MATERIALS AND METHODS

### Patients and specimens

All patients were recruited from the OHSU Knight Cancer Institute under approved institute protocols. All patients underwent ovarian cancer staging and/or debulking surgery when this was considered an effective option. Tumor subtypes were classified according to 2014 World Health Organization criteria and we utilized binary grading system [25]. Medical records of patients were retrospectively reviewed under the approved Institutional protocol (Oregon Health & Science University IRB protocol 921) that required written patient consent. All patients undergoing chemotherapy received platinum-based treatments as a first line treatment. Overall survival and progression times were determined, each measured from the time of diagnosis at initial surgery. Recurrence was defined as objective evidence of recurrence or progression by imaging studies or death. The duration of overall survival was the interval between diagnosis and death. Data were censored as of the last follow-up for patients with no evidence of recurrence, progression, or death.

### Tissue microarrays

The multiple normal tissue and breast cancer microarrays (TMA) were obtained from commercial suppliers (US Biomax, Rockville, MD, USA, Cat# MNO1021, BR1506 and BR1510; and Pantomics, Richmond, CA, USA; Cat# BRC965, BRD1021 and BRC2281).

Ovarian cancer tissue samples (188 total) were obtained from the Oregon Ovarian Cancer Registry and Tissue Repository (OOCRTR) at the Oregon Health and Science University, and paraffin-embedded and analyzed as previously described [25, 26]. Briefly, 0.6 mm cores were drawn from each block (donor blocks) and transferred to microarray blocks (receiver blocks). To overcome tumor heterogeneity, core samples were taken from three different areas of each tumor. Receiver blocks were sectioned, and individual sections were labeled with H&E, to identify the presence of tumor, or probed with an antibody to GRB7. All samples were collected with IRB approval.

### GRB7 Immunohistochemistry (paraffin tissue sections), imaging, staining classification

#### Antibody

Anti-human GRB7, a monoclonal antibody produced in rabbit, was purchased from Abcam (Cambridge, MA, USA, Cat# 183737 [EPR14099]).

TMA embedded in FFPE blocks were serially cut into 6 μm-thick sections using a microtome, directly transferred on SuperFrost Plus glass slides, and incubated for 2 h at 60° C. Slides were deparaffinized in xylene, followed by a rehydration in an ethanol series (100%, 95%, 70% and 50%) and kept in water until the next step. Antigenity was enhanced by boiling the sections in antigen retrieval buffer (10mM Tris Base, 1mM EDTA, 0.05% Tween-20, pH9) at 95° C for 20 min, followed by a step cool down (5 min at RT, 5 min cold water, 5 min ice). Endogenous peroxidases were blocked with Bloxall (Vector Labs, Burlingame, CA, USA, Cat# SP-6000) for 10 min at RT. Slides were then incubated in blocking buffer (0.5% BSA in 0.1M Tris/0.15M NaCl) for 30 min at RT, followed by incubation with the primary antibody (GRB7, 1:1500, in 0.1% BSA/0.25% Triton X-100 blocking solution) for 48 h at 4° C in a humid chamber. Slides were rinsed in 0.1M Tris/0.15M NaCl, incubated with an anti-rabbit secondary antibody coupled to peroxidase according to standard protocols with the Vectastain Elite ABD HRP kit (Vector Labs, Cat# PK-6101), and developed using the ImmPACT DAB chromogen (Vector labs, Cat# SK-4105) kit. After rinsing slides in 0.1M phosphate buffer, sections were counterstained with Hematoxylin according to standard protocols. Sections were then dehydrated (50%, 75%, 90% and 100% ethanol), cleared in xylene and mounted in Permount Mounting Medium (Thermo Fisher Scientific, Waltham, MA, USA, Cat# SP15-100). As a negative control, block sections were also labeled without exposure to primary antibody.

For image analysis, subcellular localization and expression levels were determined for both cytoplasmic and membrane fractions. The extent of immunochemical reactivity was graded on a scale of 0-3 based on intensity as follows: background (0), light (1), moderate (2), and strong (3) GRB7 stainings. The grading was performed manually by an experienced Gynecologic Oncology pathologist (P M-F). The grading was blinded to the clinical outcome or other associated biomarkers. Images were taken on an AXIO imager.A2 microscope (Zeiss, White Plains, NY, USA), attached to an Axiocam ERc 5s camera, using the ZEN2-blue software.

### Breast cancer cell line cultures

Breast cancer cell lines MDA-MB-231, HCC1954 and SKBR3 were grown in RPMI-1640 (HyClone, Logan, UT, USA, Cat# SH30027.01), supplemented with 10% Fetal Bovine Serum (Thermo Fisher Scientific, Cat# 26140–079). All cells were grown to approximately 80% confluence prior to splitting/harvesting. All breast cancer cell lines were initially obtained from ATCC.

### Western blots

#### Antibodies

anti-human GRB7, rabbit monoclonal antibody (Abcam, Cat# 183737 [EPR14099]); anti α-tubulin, mouse monoclonal antibody (Cell signaling, Danvers, MA, USA, Cat# 3873s); anti HER-2/ErbB2, rabbit polyclonal antibody (Cell signaling, Cat# 2242).

Breast and ovarian cell lines were lysed in Cell Lysys Buffer (20 mM Tris-HCl (pH 7.5), 150 mM, NaCl 1 mM, Na_2_EDTA, 1 mM EGTA, 1% Triton X-100) supplemented with HALT protease and phosphatase inhibitor cocktail (Thermo Fisher Scientific, Cat# 78442), incubated on ice for 15 min, centrifuged for 10 min at 10,000 rpm at 4° C, and the supernatant transferred to a new tube. Total protein concentration was quantified using the Pierce BCA protein assay kit (Thermo Fisher Scientific, Cat# 23225). 40 μg of protein were denatured in reducing buffer, heated to 95° C for 10 min, and run on a 7.5% gel (Bio-Rad, Hercules, CA, USA, Cat# 456–1025). Following SDS/PAGE gel electrophoresis, proteins were transferred to nitrocellulose membranes, which were probed for GRB7 (1:1000), HER-2 (1:1000) and α-tubulin (1:2000). Fluorescent protein-conjugated secondary antibodies (LI-COR Biosciences, Lincoln, NE, USA, Cat# IRDye^®^680RD-donkeyamouse and IRDye^®^800CW-donkeyarabbit) were then used for signal detection. Visualization was carried-out with the LI-COR Odyssey Infrared Imaging System Scan, with a resolution of 169 μm (LI-COR).

### Immunocytochemistry (cells in culture)

#### Antibodies

anti-human GRB7, rabbit monoclonal antibody (Abcam, Cat# 183737 [EPR14099]); goat anti-rabbit-Alexa Fluor^488^ secondary antibody (Abcam, Cat# 150077).

Cell cultures grown on Lab-Tek^®^II 8-well chamber slide systems (Thermo Fisher Scientific, Cat# 154534) were fixed for 20 min with 4% paraformaldehyde/4% sucrose in PBS at RT and followed by washes in PBS. Cells were then permeabilized with 0.3% Triton X-100 in PBS for 5 min at RT, and washed again in PBS. Slides were then incubated in blocking buffer (0.5% BSA in 0.1M Tris/0.15M NaCl) for 30 min at RT, followed by the incubation with the primary antibody (GRB7, 1:1500, in 0.1% BSA/0.25% Triton X-100 blocking solution) for 24 h at 4° C in a humid chamber. Slides were then developed in two ways: 1) IHC, using the Vectastain Elite ABD HRP kit as described above), and 2) Immunofluorescence, described here. Following three washes with PBS, cells were blocked again for 30 min, washed three more times with PBS and incubated with an anti-rabbit secondary antibody coupled to Alexa Fluor^488^. Cells were washed with PBS, and incubated for 3 min with 5μg/ml WGA-Alexa-Fluor^647^- (Thermo Fisher Scientific, Cat #W32466) and 300nM DAPI (Thermo Fisher Scientific, Cat# D1306) to label the plasma membrane and the nuclei. Following three more washes with PBS, cells were mounted using Everbrite mounting medium (Biotium, Fremont, CA, USA, Cat# 32002). Cells were imaged using an inverted epifluorescent Zeiss Axio Observer Z1 microscope, a 20× objective, and an Andor Luca EMCCD camera (Andor Technology, South Windsor, CT, USA). Fluorescence for Alexa Fluor^488^ and Alexa Fluor^647^ was detected using 485 nm excitation/535nm emission and 640 nm excitation/680 nm emission wavelengths respectively, DAPI was detected using 350 nm excitation/430 nm emission (Chroma Technologies Corp, Bellows Falls, VT, USA). In all experiments, cells were imaged at middle height of the cell, with exposure times of 200 ms. Merged images were prepared using FIJI software [27].

### Statistical analysis

Chi-squared tests were used to test for associations between GRB7 expression and clinical covariates. A multivariate logistic regression was used to identify independent prognostic factors, and a simple kappa coefficient estimate was used to test for agreement between variables. Survival curves were generated using the Kaplan-Meier method, with significance evaluated using the log-rank test of significance, and in some cases a Wilcoxon test was used as comparison. All analyses were performed with SAS software version 9.4 (SAS Institute, Cary, NC, USA), and graphs were generated by R software and Graphpad Prism.

## SUPPLEMENTARY MATERIALS



## References

[1] Shen TL, Guan JL Grb7 in intracellular signaling and its role in cell regulation. Front Biosci. 2004; 9:192–200. 10.2741/1229. 14766359

[2] Chu PY, Tai YL, Shen TL Grb7, a critical mediator of EGFR/ErbB signaling, in cancer development and as a potential therapeutic target. Cells. 2019; 8:435. 10.3390/cells8050435. 31083325 PMC6562560

[3] Tanaka S, Sugimachi K, Kawaguchi H, Saeki H, Ohno S, Wands JR Grb7 signal transduction protein mediates metastatic progression of esophageal carcinoma. J Cell Physiol. 2000; 183:411–15. 10.1002/(SICI)1097-4652(200006)183:3<411::AID-JCP14>3.0.CO;2-Z. 10797316

[4] Bai T, Luoh SW GRB-7 facilitates HER-2/Neu-mediated signal transduction and tumor formation. Carcinogenesis. 2008; 29:473–79. 10.1093/carcin/bgm221. 17916906

[5] Luoh SW, Wagoner W, Wang X, Hu Z, Lai X, Chin K, Sears R, Ramsey E GRB7 dependent proliferation of basal-like, HER-2 positive human breast cancer cell lines is mediated in part by HER-1 signaling. Mol Carcinog. 2019; 58:699–707. 10.1002/mc.22963. 30604896 PMC6561472

[6] Kauraniemi P, Bärlund M, Monni O, Kallioniemi A New amplified and highly expressed genes discovered in the ERBB2 amplicon in breast cancer by cDNA microarrays. Cancer Res. 2001; 61:8235–40. 11719455

[7] Kauraniemi P, Kuukasjärvi T, Sauter G, Kallioniemi A Amplification of a 280-kilobase core region at the ERBB2 locus leads to activation of two hypothetical proteins in breast cancer. Am J Pathol. 2003; 163:1979–84. 10.1016/S0002-9440(10)63556-0. 14578197 PMC1892409

[8] Luoh SW Amplification and expression of genes from the 17q11 approximately q12 amplicon in breast cancer cells. Cancer Genet Cytogenet. 2002; 136:43–47. 10.1016/S0165-4608(01)00657-4. 12165450

[9] Ramsey B, Bai T, Hanlon Newell A, Troxell M, Park B, Olson S, Keenan E, Luoh SW GRB7 protein over-expression and clinical outcome in breast cancer. Breast Cancer Res Treat. 2011; 127:659–69. 10.1007/s10549-010-1010-0. 20635137

[10] Luoh SW, Ramsey B, Hanlon Newell A, Troxell M, Hu Z, Chin K, Spellman P, Olson S, Keenan E HER-2 gene amplification in human breast cancer without concurrent HER-2 over-expression. Springerplus. 2013; 2:386. 10.1186/2193-1801-2-386. 24102037 PMC3791222

[11] Nadler Y, González AM, Camp RL, Rimm DL, Kluger HM, Kluger Y Growth factor receptor-bound protein-7 (Grb7) as a prognostic marker and therapeutic target in breast cancer. Ann Oncol. 2010; 21:466–73. 10.1093/annonc/mdp346. 19717535 PMC2826097

[12] Chu PY, Li TK, Ding ST, Lai IR, Shen TL EGF-induced Grb7 recruits and promotes Ras activity essential for the tumorigenicity of Sk-Br3 breast cancer cells. J Biol Chem. 2010; 285:29279–85. 10.1074/jbc.C110.114124. 20622016 PMC2937960

[13] Giricz O, Calvo V, Pero SC, Krag DN, Sparano JA, Kenny PA GRB7 is required for triple-negative breast cancer cell invasion and survival. Breast Cancer Res Treat. 2012; 133:607–15. 10.1007/s10549-011-1822-6. 22005836

[14] Sparano JA, Goldstein LJ, Childs BH, Shak S, Brassard D, Badve S, Baehner FL, Bugarini R, Rowley S, Perez EA, Shulman LN, Martino S, Davidson NE, et al Relationship between quantitative GRB7 RNA expression and recurrence after adjuvant anthracycline chemotherapy in triple-negative breast cancer. Clin Cancer Res. 2011; 17:7194–203. 10.1158/1078-0432.CCR-10-3357. 21933890 PMC3570203

[15] Wang Y, Chan DW, Liu VW, Chiu P, Ngan HY Differential functions of growth factor receptor-bound protein 7 (GRB7) and its variant GRB7v in ovarian carcinogenesis. Clin Cancer Res. 2010; 16:2529–39. 10.1158/1078-0432.CCR-10-0018. 20388850

[16] Han DC, Shen TL, Guan JL Role of Grb7 targeting to focal contacts and its phosphorylation by focal adhesion kinase in regulation of cell migration. J Biol Chem. 2000; 275:28911–7. 10.1074/jbc.M001997200. 10893408

[17] Shen TL, Han DC, Guan JL Association of Grb7 with phosphoinositides and its role in the regulation of cell migration. J Biol Chem. 2002; 277:29069–77. 10.1074/jbc.M203085200. 12021278

[18] Tsai NP, Ho PC, Wei LN Regulation of stress granule dynamics by Grb7 and FAK signalling pathway. EMBO J. 2008; 27:715–26. 10.1038/emboj.2008.19. 18273060 PMC2265756

[19] García-Palmero I, Villalobo A Calmodulin regulates the translocation of Grb7 into the nucleus. FEBS Lett. 2012; 586:1533–39. 10.1016/j.febslet.2012.04.017. 22673522

[20] Tanaka S, Pero SC, Taguchi K, Shimada M, Mori M, Krag DN, Arii S Specific peptide ligand for Grb7 signal transduction protein and pancreatic cancer metastasis. J Natl Cancer Inst. 2006; 98:491–98. 10.1093/jnci/djj105. 16595785

[21] Itoh S, Taketomi A, Tanaka S, Harimoto N, Yamashita Y, Aishima S, Maeda T, Shirabe K, Shimada M, Maehara Y Role of growth factor receptor bound protein 7 in hepatocellular carcinoma. Mol Cancer Res. 2007; 5:667–73. 10.1158/1541-7786.MCR-06-0282. 17634422

[22] Zhao HB, Zhang XF, Jia XL, Wang HB Grb7 is over-expressed in cervical cancer and facilitate invasion and inhibit apoptosis in cervical cancer cells. Pathol Res Pract. 2017; 213:1180–84. 10.1016/j.prp.2017.05.013. 28780081

[23] Chan DW, Hui WW, Cai PC, Liu MX, Yung MM, Mak CS, Leung TH, Chan KK, Ngan HY Targeting GRB7/ERK/FOXM1 signaling pathway impairs aggressiveness of ovarian cancer cells. PLoS One. 2012; 7:e52578. 10.1371/journal.pone.0052578. 23285101 PMC3527599

[24] Nelson AC, Machado HL, Schwertfeger KL Breaking through to the Other Side: Microenvironment Contributions to DCIS Initiation and Progression. J Mammary Gland Biol Neoplasia. 2018; 23:207–21. 10.1007/s10911-018-9409-z. 30168075 PMC6237657

[25] Kononen J, Bubendorf L, Kallioniemi A, Bärlund M, Schraml P, Leighton S, Torhorst J, Mihatsch MJ, Sauter G, Kallioniemi OP Tissue microarrays for high-throughput molecular profiling of tumor specimens. Nat Med. 1998; 4:844–47. 10.1038/nm0798-844. 9662379

[26] Wysham WZ, Mhawech-Fauceglia P, Li H, Hays L, Syriac S, Skrepnik T, Wright J, Pande N, Hoatlin M, Pejovic T BRCAness profile of sporadic ovarian cancer predicts disease recurrence. PLoS One. 2012; 7:e30042. 10.1371/journal.pone.0030042. 22253870 PMC3256213

[27] Schindelin J, Arganda-Carreras I, Frise E, Kaynig V, Longair M, Pietzsch T, Preibisch S, Rueden C, Saalfeld S, Schmid B, Tinevez JY, White DJ, Hartenstein V, et al Fiji: an open-source platform for biological-image analysis. Nat Methods. 2012; 9:676–82. 10.1038/nmeth.2019. 22743772 PMC3855844

