# Supplementary material for: The prognostic significance of GRB7 protein expression and localization in human breast and ovarian cancers
Source: Oncotarget. 2020 Jun 16;11(24):2273–89. doi: 10.18632/oncotarget.27593 (PMC7299530; doi:10.18632/oncotarget.27593)
Supplement: Supplementary file 1 [file oncotarget-11-2273-s001.pdf]

The prognostic significance of GRB7 protein expression and localization in human breast and ovarian cancers

SUPPLEMENTARY MATERIALS

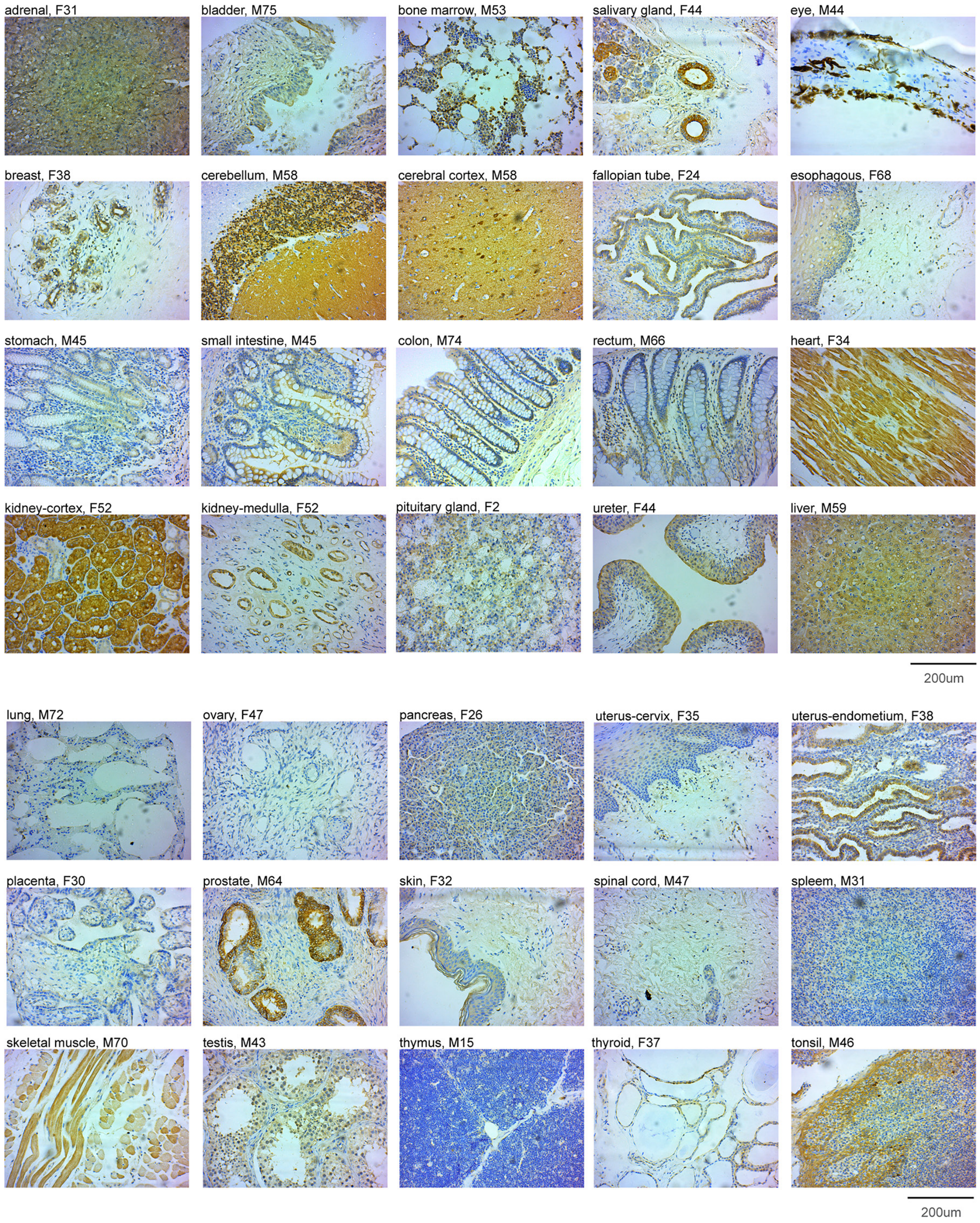

Supplementary Figure 1: GRB7 expression in normal human tissues. Examples of tissues from multi-organ tissue microarrays (TMAs) stained with the GRB7 antibody (brown) and Hematoxylin (blue). The type of tissue is indicated at the top of each image, followed by gender (F female, M male) and age (years) of the tissue donor. Images were taken with a 20 × objective, scale bar is 200 um.

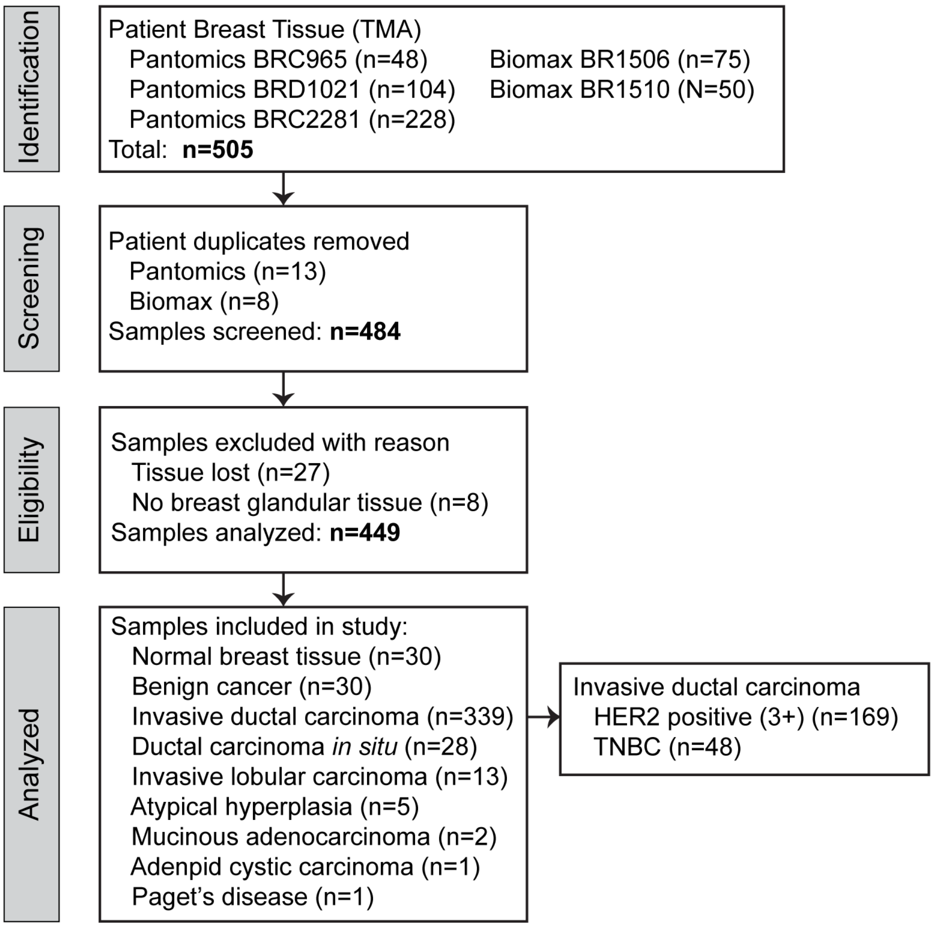

Supplementary Figure 2: Flow chart showing selection and characteristic of breast tissue samples analyzed.

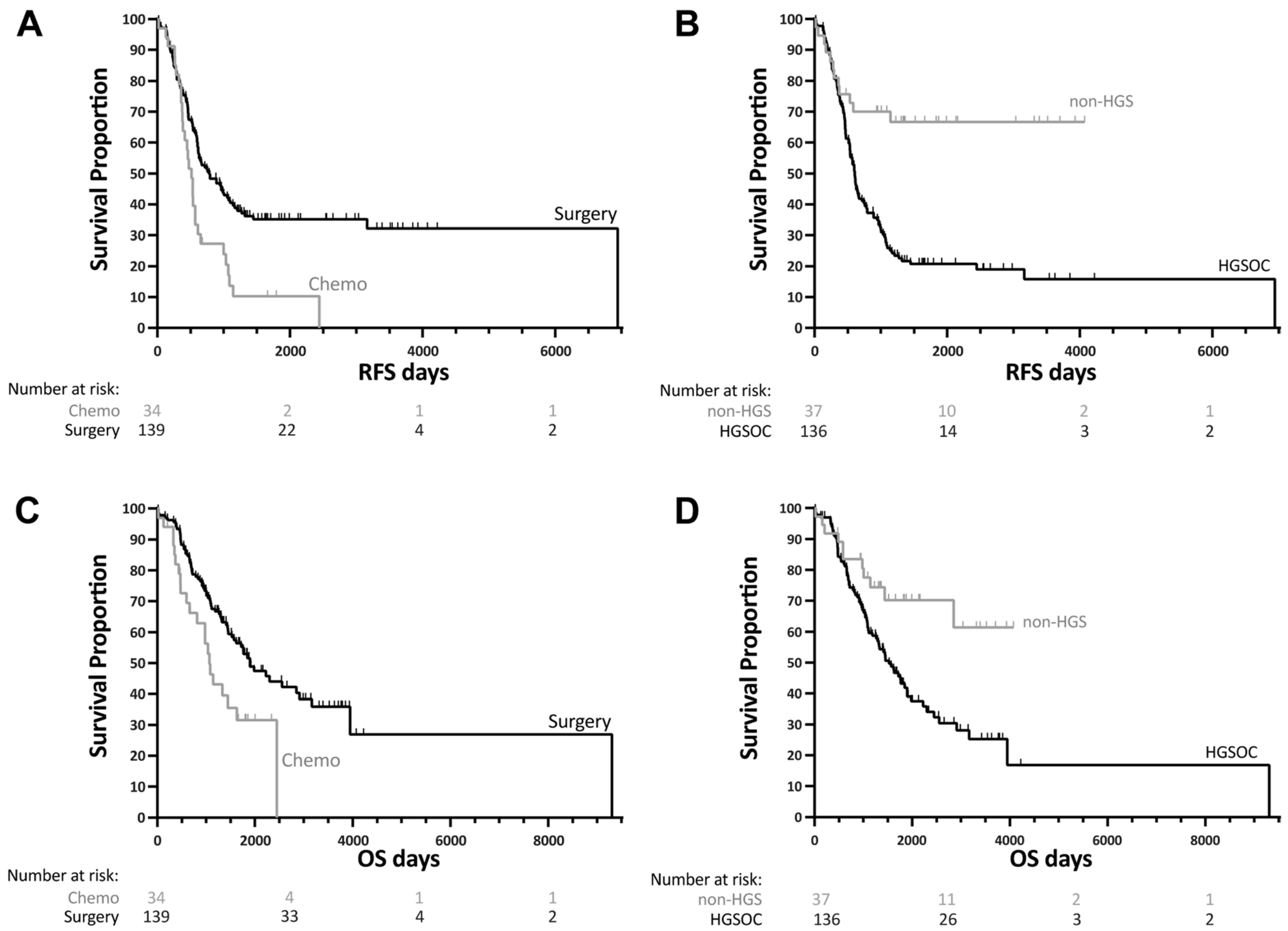

Supplementary Figure 3: Kaplan-Meier survival curves for ovarian cancer-free intervals for primary cancer treatment and histology. Recurrence Free Survival (RFS, top) and Overall Survival (OS, bottom) of cohort patients with ovarian cancer by primary treatment and histology. (**A**, **C**) neo-adjuvant (gray curves, chemo) vs. adjuvant (black curves, surgery) chemo. (**B**, **D**) non-HGS (gray curves, non-high grade serous ovarian cancer) vs. HGSOC (black curves, high grade serous ovarian cancer).

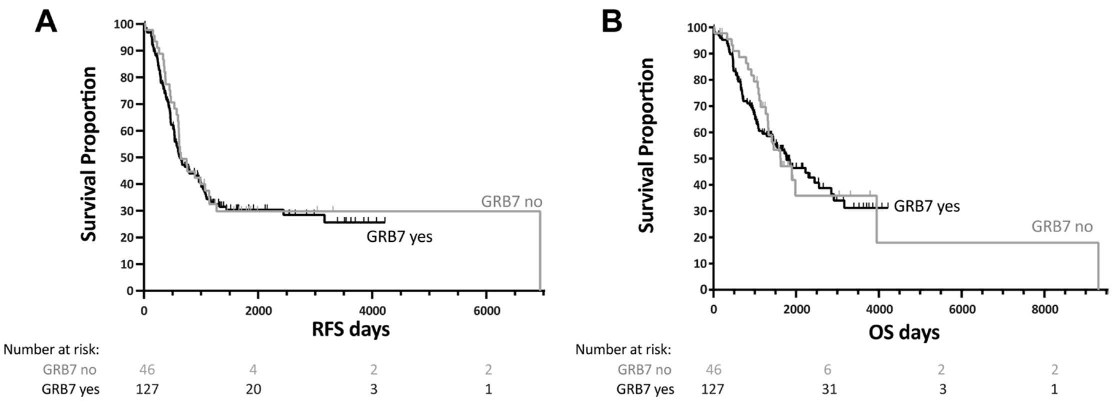

Supplementary Figure 4: Kaplan-Meier survival curves for cytoplasmic GRB7 staining. Cytoplasmic GRB7 expression (largest GRB7 expression value of the cores for each patient, black curves, GRB7 yes) *vs.* no GRB7 expression (gray curves, GRB7 no) has no significantly effects on (**A**) Recurrence Free Survival (RFS) nor (**B**) Overall Survival (OS) of patients. All survival curves were generated after dichotomization of GRB7 cytoplasmic expression or no GRB7 cytoplasmic expression, obtained by IHC. *p*-values were calculated by log-rank test.

Supplementary Table 1: Clinical parameters analyzed of the 173 patients included in the study

		N	mean	median	StdDev	Min	Max	95% CI (for mean)
Primary Treatment	Neo-adjuvant Chemo	34	64.29	64.5	12.24	27	56	(60.02–68.56)
	Adjuvant chemo	137[b]	60.36	61	12.24	20	69	(58.29–62.43)
Histology Type	Non-HGS	35[b]	58.11	60	12.37	24	52	(53.86–62.36)
	HGSOC	136	61.92	63	12.22	20	69	59.85–63.99)
GRB7 Expression	0–1+	101[a]	59.75	60	13.36	24	89	(57.12–62.39)
	2–3+	70[a]	63.14	64	10.38	20	84	(60.67–65.62)
	**Total**	171	61.14	62	12.31	20	89	(59.28–63.00)

Relationship between the age of the patient at diagnosis of ovarian cancer and 1) primary treatment (neo-adjuvant chemo vs. adjuvant chemo), 2) histology type (non-HGS or HGSOC), and 3) GRB7 expression (shown as the maximum value of the available cores / patient). non-HGS = non-high grade serous ovarian cancer, HGSOC = high grade serous ovarian cancer. (a) 1 data point missing for the group, (b) 2 data points missing for the group.

Supplementary Table 2: Clinical events analyzed for ovarian cancer patients

Status	Total	Recurrence free survival		Overall survival	
		censored	event	censored	event
AWD (alive with disease)	35	11	24	35	0
Alive	2	2	0	2	0
DOD (dead of disease)	91	0	91	0	91
NED (no evidence of disease)	44	41	3	44	0
Dead from cerebral palsy	1	0	1	0	1
Total	173	54	119	81	92

Supplementary Table 3: Recurrence Free Survival (RFS) and Overall Survival (OS) of cohort patients with ovarian cancer

A		Recurrence free survival (RFS)				Median survival			Mean survival		Log-rank
		Total	Event	Censored		Estimate	Lower 95% CI	Upper 95% CI	Mean	SD	Test *p*-value
all		173	119	54	31.2	648	575	947	2323.23	239.99	
Primary Treatment	Neo-adjuvant	34	30	4	11.76	516.00	381.00	575.00	726.74	116.32	**0.0011**
	adjuvant	139	89	50	35.97	785.00	609.00	1077.00	2688.65	277.74	
Histology	Non-HGS	37	12	25	67.57				886.64	69.91	**<0.0001**
	HGSOC+	136	107	29	21.32	609.00	533.00	716.00	1692.34	235.48	

B		Overall survival (OS)				Median survival			Mean survival		Log-rank
		Total	Event	Censored		Estimate	Lower 95% CI	Upper 95% CI	Mean	SD	Test *p*-value
all		173	92	81	46.82	1758.00	1411.00	2299.00	3427.58	492.31	
Primary Treatment	Neo-adjuvant	34	22	12	35.29	1070.00	660.00	1628.00	1306.93	157.23	**0.0066**
	adjuvant	139	70	69	49.64	1899.00	1527.00	2909.00	3707.65	548.17	
Histology	Non-HGS	37	11	26	70.27		2849.00		2212.36	180.95	**0.0066**
	HGSOC+	136	81	55	40.44	1527.00	1184.00	1899.00	2844.11	485.55	

(A) RFS and OS based on primary treatment (neo-adjuvant vs. adjuvant chemo). (B) RFS and OS based on histology (non-HGS vs. HGSOC).

Supplementary Table 4: No significant correlation between cytoplasmic GRB7 staining and RFS nor OS was detected in patients with ovarian cancer

A		# of events				Median survival			Mean survival		Log-rank
		Total	Event	Censored		Estimate	Lower 95% CI	Upper 95% CI	Mean	SD	Test *p*-value
	0	46	31	15	32.61	648.00	575.00	1140.00	2487.70	458.50	
RFS	1,2,3+	127	88	39	30.71	630.00	533.00	966.00	1316.63	112.35	0.6687
	all	173	119	54	31.21	648	575	947	2323.23	239.99	
OS	0	46	25	21	45.65	1628.00	1309.00	3945.00	3148.59	853.71	
	1,2,3+	127	67	60	47.24	1763.00	1292.00	2554.00	1864.60	110.39	0.7850
	all	173	92	81	46.82	1758.00	1411.00	2299.00	3427.58	492.31	

B		# of events				Median survival			Mean survival		Log-rank
		Total	Event	Censored		Estimate	Lower 95% CI	Upper 95% CI	Mean	SD	Test *p*-value
	0	46	31	15	32.61	648.00	575.00	1140.00	2487.70	458.50	
	1+	56	40	16	28.57	607.50	468.00	967.00	781.36	69.15	
RFS	2+	33	24	9	27.27	600.00	433.00	1077.00	709.83	71.09	0.7484
	3+	38	24	14	36.84	756.50	517.00	3164.00	1546.59	218.93	
	all	173	119	54	31.2	648	575	947	2323.23	239.99	
	0	46	25	21	45.65	1628.00	1309.00	3945.00	3148.59	853.71	
OS	1+	56	27	29	51.79	2223.00	1184.00		1900.41	152.11	
	2+	33	19	14	42.42	1292.00	663.00		1190.03	106.45	0.7166
	3+	38	21	17	44.74	1899.00	914.00	3164.00	1861.47	202.69	
	all	173	92	81	46.82	1758.00	1411.00	2299.00	3427.58	492.31	

C		# of events				Median survival			Mean survival		Log-rank
		Total	Event	Censored		Estimate	Lower 95% CI	Upper 95% CI	Mean	SD	Test *p*-value
	0 – 2+	135	95	40	29.63	630.00	540.00	967.00	2299.14	257.03	
RFS	3+	38	24	14	36.84	756.50	517.00	3164.00	1546.59	218.93	0.4190
	all	173	119	54	31.21	648	575	947	2323.23	239.99	
OS	0 – 2+	135	71	64	47.41	1758.00	1330.00	2299.00	3369.80	605.81	
	3+	38	21	17	44.74	1899.00	914.00	3164.00	1861.47	202.69	0.8321
	all	173	92	81	46.82	1758.00	1411.00	2299.00	3427.58	492.31	

(A) GRB7 expression in the cytoplasm does not significantly change RFS nor OS. (B) Strong expression of cytoplasmic GRB7 (3+) is not significantly different when compared to medium (2+), weak (1+) or no GRB7 staining. (C) Strong expression of cytoplasmic GRB7 (3+) is not significantly different when compared to less than strong (0 to 2+) GRB7 staining.
